# Supplementary material for: The interactome of intact mitochondria by cross-linking mass spectrometry provides evidence for coexisting respiratory supercomplexes
Source: Mol Cell Proteomics. 2017 Dec 8;17(2):216–32. doi: 10.1074/mcp.RA117.000470 (PMC5795388; doi:10.1074/mcp.RA117.000470)
Supplement: Supplemental Data [file supp_RA117.000470_133922_0_supp_23978_fzffwf.zip › spectra_annotation/mito_spectra_annotation/405-2-3-1-3-1.pdf]

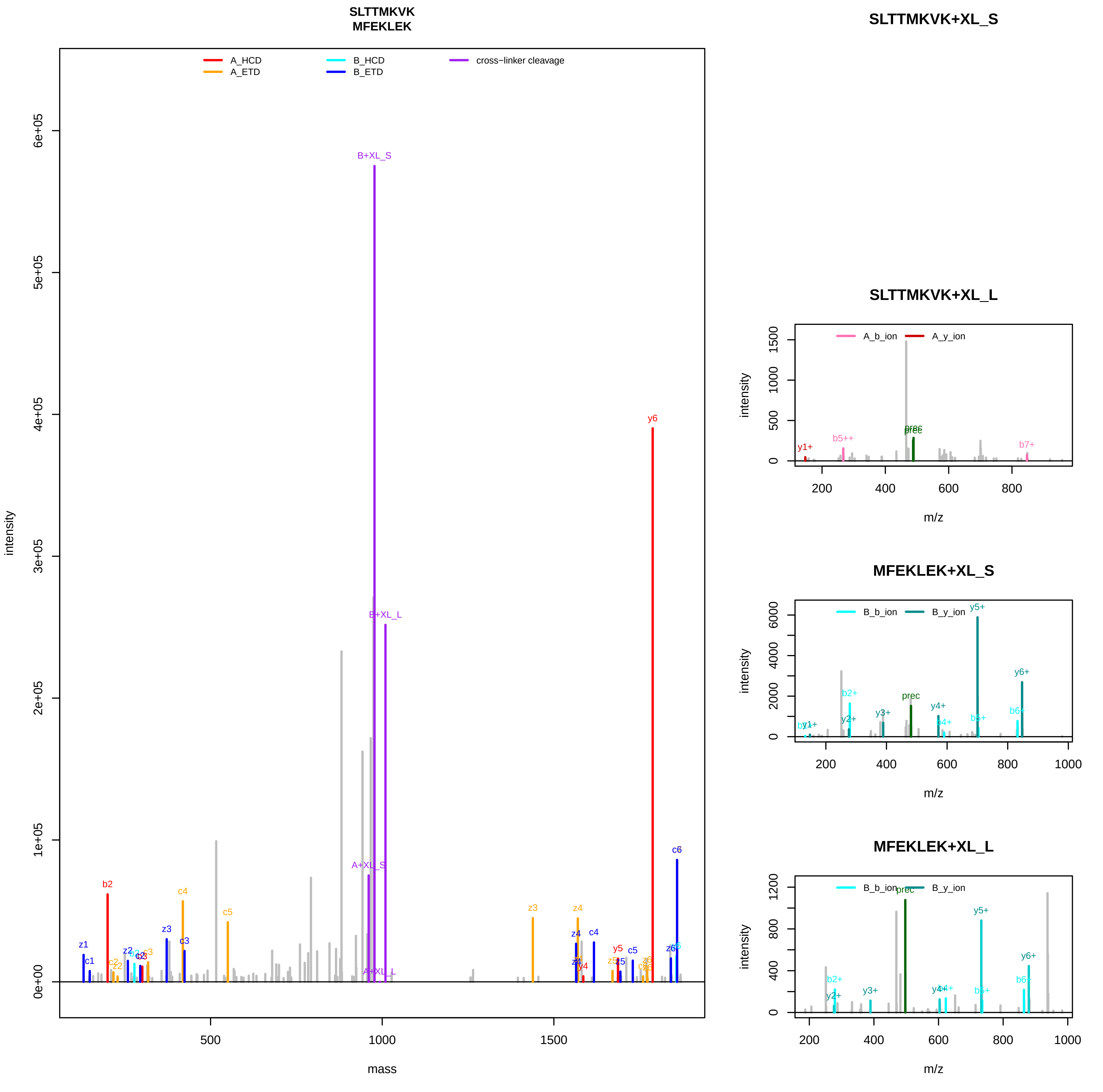

SLTTMKVK
MFEKLEK
A_HCD
A_ETD
B_HCD
B_ETD
cross−linker cleavage
B+XL_S
B+XL_L
A+XL_S
A+XL_L
y6
c6
b2
c4
c5
z3
z4
y5
c5
y4
z1
c1
z2
c2
c3
z3
z4
c4
z5
z6
intensity
mass
0e+00
1e+05
2e+05
3e+05
4e+05
5e+05
6e+05
500
1000
1500
SLTTMKVK+XL_S
SLTTMKVK+XL_L
A_b_ion
A_y_ion
y1+
b5++
prec
b7+
intensity
m/z
MFEKLEK+XL_S
B_b_ion
B_y_ion
y5+
y6+
b2+
y2+
y3+
prec
y4+
b4+
b5+
b6+
y1+
MFEKLEK+XL_L
B_b_ion
B_y_ion
prec
y5+
y6+
b2+
y2+
y3+
y4+
b5+
b6+
